# Detection of Testicular Metastasis from Renal Cell Carcinoma on PSMA-PET Scan

**DOI:** 10.15586/jkcvhl.v11i1.268

**Published:** 2024-03-05

**Authors:** Theresa Olmstead, Michael Emmerling, Surekha Bantumilli, Mathew Raynor, Matthew E. Nielsen, Marc A. Bjurlin, Tracy L. Rose

**Affiliations:** 1 University of Washington School of Medicine, Seattle, WA;; 2 Department of Urology, University of North Carolina at Chapel Hill, Chapel Hill, NC;; 3 Department of Pathology, University of North Carolina at Chapel Hill, Chapel Hill, NC;; 4 Lineberger Comprehensive Cancer Center, University of North Carolina at Chapel Hill, Chapel Hill, NC;; 5 Division of Oncology, Department of Medicine, University of North Carolina at Chapel Hill, Chapel Hill, NC

**Keywords:** PET imaging, RCC, prostate cancer

## Abstract

The use of prostate-specific membrane antigen–positron emission tomography (PSMA-PET) is becoming more widespread for the diagnosis and management of prostate cancer. Here we report a case of oligometastatic renal cell carcinoma (RCC) to the testes diagnosed incidentally on PSMA-PET imaging. This case demonstrates the potential for diagnosis of nonprostate disease with PSMA-PET imaging, as well as the promising nature of PSMA-PET for the diagnosis and surveillance of RCC. In addition, this case report discusses the rare occurrence of oligometastatic RCC to the testis.

## Introduction

In this report, we present a patient with an oligometastatic recurrence of renal cell carcinoma (RCC) discovered incidentally on prostate-specific membrane antigen–positron emission tomography (PSMA-PET) scan during the workup of biochemically recurrent prostate cancer. Despite its name, PSMA expression is not restricted to the prostate (1). PSMA is also expressed in the neovasculature of solid tumors, including the highly vascular tumors of RCC. Higher levels of PSMA expression by immunohistochemistry (IHC) are associated with worse outcomes in clear cell RCC (2). PSMA-PET is therefore being investigated in the staging and restaging of RCC with early studies suggesting that it may outperform conventional computerized tomography (CT) imaging (3).

## Case Report

A 68-year-old male presented with a renal mass discovered incidentally on ultrasound during workup of elevated liver function tests. Primary staging was performed with contrast-enhanced CT and showed a 5 cm enhancing upper pole left renal mass with no evidence of metastatic disease. He underwent an uncomplicated left radical nephrectomy and surgical pathology revealed a grade 3, 4.5 cm clear cell RCC, pT3aN0, with extension into the renal sinus and renal vein with negative surgical margins. One lymph node was removed and negative for cancer. Surveillance imaging with CT at 6-months post-nephrectomy was without evidence of recurrence.

Approximately 7 months after his RCC diagnosis, his prostate-specific antigen (PSA) was elevated at 4.3 ng/mL on routine screening. This prompted a multi-parametric prostate magnetic resonance imaging (MRI) demonstrating a PIRADS 5 region of interest. Subsequent MRI-ultrasound (US) fusion prostate biopsy confirmed Gleason Grade Group 4 prostate adenocarcinoma. He underwent a radical prostatectomy. Surgical pathology revealed Gleason 4+3=7 pT2N0 (none out of eight nodes) prostate adenocarcinoma with intraductal carcinoma (70% Gleason 4) with negative margins. PSA at 4 months postoperatively was undetectable. Subsequent PSA at 8 months was 2.2 ng/mL, consistent with biochemical recurrence.

**Figure 1:**
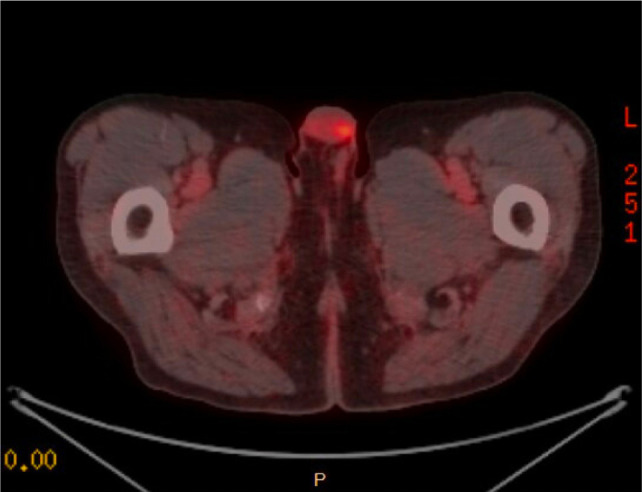
PSMA-PET demonstrating increased uptake in the right testicle, concerning for metastasis.

**Figure 2:**
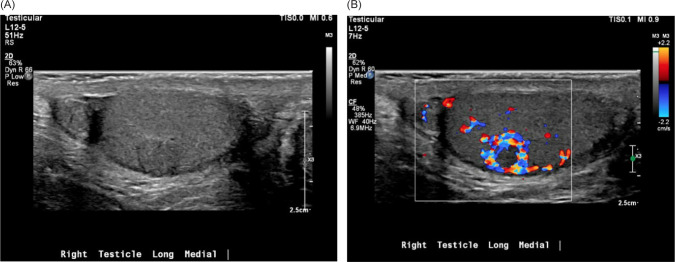
(A) Scrotal ultrasound demonstrating long view of the medial right testicle with echogenic, heterogeneous, and well-circumscribed mass. (B) Doppler ultrasound demonstrating hypervascularity of the previously characterized mass.

**Figure 3:**
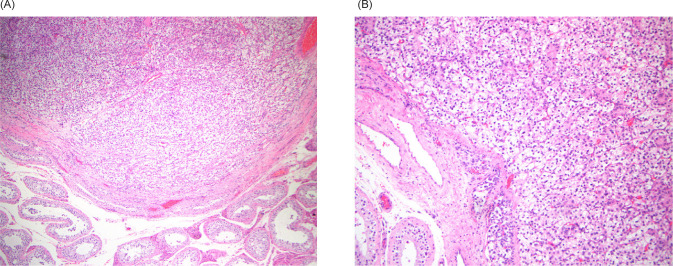
(A) Pushing border of well-circumscribed tumor in testis (4×); (B) Tumor shows classic well-vascularized nests of cells with clear cytoplasm (10×).

**Figure 4:**
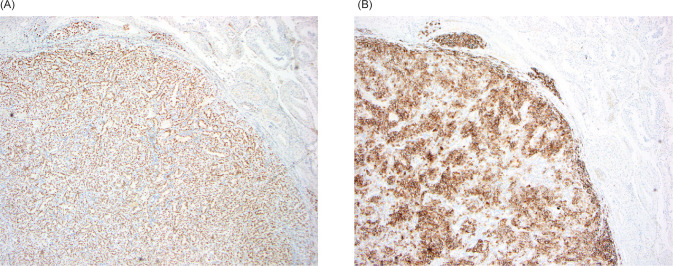
(A) Strong nuclear expression of PAX8 consistent with renal origin; (B) Strong membrane expression of CAIX supportive of clear cell RCC.

A PSMA–PET/CT scan was performed and was notable for a PET-avid right testicular mass (Figure 1). Subsequent scrotal ultrasound showed a solid, hypervascular 8 mm × 7 mm × 7 mm mass in the right testicle concerning for primary germ cell tumor (Figure 2). Serum tumor markers (AFP, LDH, and HCG) were obtained which were unremarkable. A right radical orchiectomy (Figure 3) was performed with final pathology demonstrating a metastatic clear cell RCC to the testis with lymphovascular invasion (Figure 4).

The patient underwent salvage prostate radiation with androgen deprivation therapy for biochemical recurrence of prostate cancer. His PSA was undetectable at 14 months postoperatively. A complete work-up of his oligometastatic clear cell RCC demonstrated no additional sites of metastasis on brain MRI, bone scan, and CT C/A/P at 7 months postoperatively. He is currently undergoing surveillance with serial scans every 3–4 months.

## Discussion

PSMA is a type II transmembrane protein encoded by the *FOLH1* gene on chromosome 11. It was originally discovered in prostatic epithelial tissue and found to be upregulated in prostate cancer (4). Despite its name, PSMA expression can be found in other benign and cancerous tissues (1). In contrast to its expression in prostatic epithelium, various other solid organ tumors show increased expression in the vascular endothelium, suggesting a potential role of PSMA in angiogenesis (5). With the increased use of PSMA-PET imaging in prostate cancer, the detection of incidental nonprostatic synchronous primary or metastatic PSMA-avid cancers has been on the rise, including renal, colorectal, gastric, lung cancers, and multiple myeloma (6).

The management of RCC is dependent on stage at diagnosis and typically determined by imaging. Current clinical practice guidelines recommend the use of contrast-enhanced CT in staging and restaging of RCC. However, contrast-enhanced CT is contraindicated in some patients due to contrast allergies or chronic kidney disease (CKD). Furthermore, smaller metastatic lesions and lymph nodes can be difficult to identify on CT. MRI is therefore an option for staging imaging, though use of contrast is still limited in patients with severe CKD. ^18^F-FDG PET is another option for staging imaging; however, it is limited in RCC due to poor sensitivity and heterogeneous uptake in both primary and metastatic RCC lesions as well as its renal excretion making it difficult to discriminate primary lesions (3). PSMA-PET may therefore be a viable alternative to conventional staging imaging for patients with conditions precluding standard contrasted CT or MRI, or in those with indeterminate imaging findings.

Our case report highlights several teaching points. First, there may be a role for PSMA-PET in staging and re-staging RCC with multiple early studies showing promise. In a large retrospective tumor analysis from archived RCCs in treatment-naïve individuals, Spatz et al. demonstrated that greater than 80% of clear cell RCCs overexpress PSMA in the endothelium with a statistically significant association between intensity of expression and grade, stage, and overall survival (2). There are several small, prospective pilot studies that demonstrate increased sensitivity of PSMA-PET compared to conventional imaging in the staging and restaging of RCC (7–9). In a retrospective case series from Australia comparing PSMA-PET and conventional imaging, management was changed in approximately half of patients as a direct result of PSMA-PET findings when compared to conventional imaging (10).

Second, given that the inhibition of angiogenesis with VEGFR-targeted therapy is highly effective for the treatment of advanced or metastatic RCC, the potential to target PSMA in the neovasculature of RCC with theranostics is intriguing. Using PSMA as a proxy for intratumor neovascularization, a hallmark of clear cell RCC, there is a theoretical opportunity for pretreatment PSMA-PET scans to help identify patients more likely to respond to antiangiogenic therapies while also avoiding systemic toxicity in those less likely. In addition, there may be prognostic value of PSMA-PET in determining the aggressiveness of tumors as suggested by Spatz et al. and supported by Gao et al., which showed statistically significant associations of SUVmax with grade, stage, and pathologic features (2,11). Lastly, there may be a role in monitoring treatment response as traditional assessments relying on size and enhancement changes are not always reliable with modern targeted and immune therapies. Mittlmeier et al. found large discordant findings in metastatic RCC patients receiving systemic therapy at the 8-week mark, with only two out of eleven patients with concordant findings based on standard response criteria on CT and PSMA-PET (12). Taken together, there could be significant diagnostic, prognostic, and therapeutic value of using PSMA-PET scans in patients with RCC.

Our case report also highlights the unique metastatic landing sites of RCC. RCC most commonly metastasizes to the lymph nodes, lung, liver, bone marrow, and brain (13). Rarely, RCC can metastasize to the testes. In general, secondary testicular tumors are quite rare (14), perhaps due to the decreased temperature and inhabitable nature of the scrotum. With a predominance of left-sided, ipsilateral testicular RCC metastasis, it has been suggested that the route of spread is retrograde via the valveless renal vein draining into the gonadal vein. However, there are also reports of both ipsilateral and contralateral right-sided metastatic lesions, questioning this theory. Camerini and Andrea et al. suggested arterial spread as a possible source, evidenced by local vascular invasion seen on histology for an ipsilateral right testicular metastasis (15). The blood–testes barrier formed by Sertoli cells to protect spermatogenesis could also play a role in protecting the testes from secondary tumors. Similar to the central nervous system and eyes, the testicles are immune privileged and considered to be a “tumor sanctuary.” Not surprisingly, this could also explain why testicular metastasis is notoriously difficult to treat with systemic therapy as penetration of drugs is often suboptimal (15). Interestingly, a testicular metastasis in a patient receiving immunotherapy with IL2 for widespread metastatic RCC showed an opposite clinical behavior (i.e. tumor progression) compared to their other metastasis sites (lymph nodes, lung, and bone marrow) (15).

## Conclusion

PSMA-PET is a molecular imaging modality that has changed the diagnosis and treatment of prostate cancer. Its increasing and widespread use in prostate cancer has shed light on its potential utility in diagnosing and managing other malignancies, such as RCC and colorectal cancer; several studies investigating its role in alternate diseases have already suggested this. Future large-scale prospective studies are needed to investigate the use of PSMA-PET in RCC. While no imaging modality, including PSMA-PET, is without limitations, additional modes of imaging could allow for more effective diagnosis, management, and surveillance of RCC patients going forward.
